# Initiatives addressing precarious employment and its effects on workers’ health and well-being: a protocol for a systematic review

**DOI:** 10.1186/s13643-021-01728-z

**Published:** 2021-06-30

**Authors:** Virginia Gunn, Carin Håkansta, Emilia Vignola, Nuria Matilla-Santander, Bertina Kreshpaj, David H. Wegman, Christer Hogstedt, Emily Q. Ahonen, Carles Muntaner, Sherry Baron, Theo Bodin, The Precarious Work Research (PWR) Group

**Affiliations:** 1 Unit of Occupational Medicine, Institute of Environmental Medicine, Karolinska Institutet, Stockholm Region, Sweden; 2 MAP Centre for Urban Health Solutions, Li Ka Shing Knowledge Institute, Unity Health Toronto, Toronto, ON Canada; 3 Lawrence S. Bloomberg Faculty of Nursing, University of Toronto, Toronto, ON Canada; 4 Working Life Science, Karlstad University, Karlstad, Sweden; 5 CUNY Graduate School of Public Health & Health Policy, New York, USA; 6 University of Massachusetts, Lowell, USA; 7 Richard M. Fairbanks School of Public Health, Indiana University, Indianapolis, IN USA; 8 Dalla Lana School of Public Health, University of Toronto, Toronto, Canada; 9 Barry Commoner Center for Health and the Environment, Queens College, City University of New York, New York, USA

**Keywords:** Precarious employment, Informal work, Decent work, Workplace, Initiative, Health outcomes, Health inequities, Public health, Systematic review, Narrative synthesis

## Abstract

**Background:**

Precarious employment is a significant determinant of population health and health inequities and has complex public health consequences both for a given nation and internationally. Precarious employment is conceptualized as a multi-dimensional construct including but not limited to employment insecurity, income inadequacy, and lack of rights and protection in the employment relation, which could affect both informal and formal workers. The purpose of this review is to identify, appraise, and synthesize existing research on the effectiveness of initiatives aiming to or having the potential to eliminate, reduce, or mitigate workers’ exposure to precarious employment conditions and its effects on the health and well-being of workers and their families.

**Methods:**

The electronic databases searched (from January 2000 onwards) are Scopus, Web of Science Core Collection, and PubMed, along with three institutional databases as sources of grey literature. We will include any study (e.g. quantitative, qualitative, or mixed-methods design) evaluating the effects of initiatives that aim to or have the potential to address workers’ exposure to precarious employment or its effects on the health and well-being of workers and their families, whether or not such initiatives were designed specifically to address precarious employment. The primary outcomes will be changes in (i) the prevalence of precarious employment and workers’ exposure to precarious employment and (ii) the health and well-being of precariously employed workers and their families. No secondary outcomes will be included. Given the large body of evidence screened, the initial screening of each study will be done by one reviewer, after implementing several strategies to ensure decision-making consistency across reviewers. The screening of full-text articles, data extraction, and critical appraisal will be done independently by two reviewers. Potential conflicts will be resolved through discussion. Established checklists will be used to assess a study’s methodological quality or bias. A narrative synthesis will be employed to describe and summarize the included studies’ characteristics and findings and to explore relationships both within and between the included studies.

**Discussion:**

We expect that this review’s findings will provide stakeholders interested in tackling precarious employment and its harmful health effects with evidence on effectiveness of solutions that have been implemented to inform considerations for adaptation of these to their unique contexts. In addition, the review will increase our understanding of existing research gaps and enable us to make recommendations to address them. Our work aligns with the sustainable development agenda to protect workers, promote decent work and economic growth, eliminate poverty, and reduce inequalities.

**Systematic review registration:**

PROSPERO CRD42020187544.

**Supplementary Information:**

The online version contains supplementary material available at 10.1186/s13643-021-01728-z.

## Background

Precarious employment is a significant determinant of population health and health inequities and has complex public health consequences both for a given nation and internationally.

The rising rates of precarious employment reported in recent decades across the globe have been brought into acute focus by the current COVID-19 pandemic, which has unearthed complex health, social, and economic vulnerabilities of workers who lack decent work. The concept of precarious employment has been explored through different research perspectives. In public health and in social and occupational epidemiology, this concept has been defined as a multi-dimensional construct including but not limited to employment insecurity, income inadequacy, and lack of rights and protection in the employment relation, which could affect both informal and formal workers [1–3].

Precarious employment is associated with a range of health problems including both mental and physical illnesses, occupation-specific afflictions, harmful life-style behaviours, and social disadvantage [4, 5]. Precarious employment is not evenly distributed in a population but rather tends to be concentrated along lines of the intersecting categories [5] of socially created disadvantage which may exist in any society, such as such as race, gender [6–8], age [9, 10], education [11], income, class [12, 13], citizenship [14], immigration status [15–17], and disability [18]. As a result, certain categories of workers, including women, youth, racialized, ethnic, or other minority subgroups, foreign-born, lower-educated, disabled, and informal workers, are exposed to higher levels of precarious employment, which, in turn, unfairly increases their risk of related health problems.

### Brief overview of existing literature

The existing literature on precarious employment addresses a broad range of topics. For instance, several studies and reports set the context for this field of research either through defining the concepts of precarious, non-standard, flexible, contingent, and informal employment [1, 2, 19–25] or through a focus on operationalization, data collection and measurement considerations, measurement tools, and identifying research priorities [3, 15, 26–42]. For instance, a range of tools have been created and validated for different national contexts to measure exposure to precarious employment, including the Spanish Employment Precariousness Scale (EPRES) [26, 27] and the Employment Precarious Index (EPI) [15, 28]. A number of studies scrutinize the socio-economic and political contexts that enable, maintain, or precipitate the spread of precarious employment [5, 21, 38, 43, 44], further laying the foundation for an in-depth understanding of precarious employment. A thorough understanding of the factors contributing to precarious employment is instrumental in the development of solutions by international [45], national, and local organizations and other stakeholders who play key roles in the improvement of working conditions.

The body of literature investigating the links between various aspects of precarious employment and health has grown considerably in recent years, as researchers work to keep up with the rampant increase in its occurrence. Although precarious employment is not always described as such, research on this topic examines its impact on a wide range of health problems including occupational injuries [4, 46–48]; musculoskeletal disorders [8, 49, 50]; poor self-rated health [49, 51, 52]; diminished well-being [53]; mental disorders and suicide [8, 18, 54–62]; cardiovascular disease [63, 64]; kidney injury [65–67]; liver disease [8]; metabolic syndrome [68]; infectious diseases, respiratory problems, and allergies [50]; harmful health behaviours [8, 69]; cancer [70, 71]; eye injuries [72]; and irregular menstruation [73]. Moreover, the impact of precarious employment on health inequities constitutes another significant research area [59, 74–80]. In addition, several authors have examined the complex effects on health of cycling in and out of precarious employment, given that many workers do not experience precarious employment in a static way at one point in time, but rather may periodically move in and out of such unfavourable work arrangements [22, 57–59].

Building on primary studies, several reviews and review protocols have been published or registered to date on topics related to precarious employment. For instance, numerous reviews consider the impact of various aspects of precarious employment on occupational accidents and injuries [81] and on health problems and diminished well-being [38, 82–92]. Other reviews aim to enrich understanding of the ways in which health inequities are exacerbated by unequal employment and working conditions across populations [93–95] and the effects that macro-level policies have on ameliorating such inequities [74, 96–98].

Several reviews and protocols focused on workplace initiatives to improve workers’ health are included in this brief review of the literature although they do not focus specifically on precariously employed workers. Given that precarious employment is associated with a range of health problems including both mental and physical illnesses, occupation-specific afflictions, and harmful life-style behaviours, workplace initiatives addressing such problems have the potential to benefit workers in precarious employment even when not targeting those workers directly. In addition, since socio-economic deprivation may prevent precariously employed workers from accessing health and social services on their own, having access to them through the workplace increases their chances of using them. As a result, the findings and recommendations from the reviews included in this section could be used to mitigate the effects of precarious employment on workers’ health and well-being in situations in which eliminating precarious employment is not possible. For instance, several reviews examine strategies and resources adopted to prevent occupational diseases and promote worker health and well-being [74, 99–111]. Some reviews study the effectiveness of improved working conditions, job re-design, and ergonomics practices on health and well-being even when workers remain precariously employed [47, 112, 113]. Others investigate the effects of employment training and coaching on: (a) labour market outcomes such as worker skills, employment status and earnings, and economic and business performance [114–116] and (b) employment outcomes for workers with disabilities [117].

Our literature search identified only two reviews that approach the scope of ours. One examined strategies adopted by trade unions to increase membership which, in turn, would positively impact bargaining power to improve employment conditions [118], while the other aimed to clarify if minimum wage rules could be used effectively to stimulate decent work and ease poverty [119]. No reviews to date show explicit attention to research that identifies solutions that could specifically counteract precarious employment and its effects on health and well-being.

### Rationale for study and contribution to the field

As highlighted in the brief overview of existing literature section, scholarship preoccupied with examining the effects of precarious employment conditions on the health and well-being of workers is steadily expanding in both volume and complexity. However, currently, there is quite limited knowledge regarding initiatives that could be adopted to counteract precarious employment and its harmful effects on health and well-being. Our systematic review intends to address this knowledge gap by identifying, evaluating, and synthesizing relevant initiatives that could be used to eliminate, reduce, or mitigate workers’ exposure to precarious employment conditions and its effects on the health and well-being of workers and their families.

In addition, our work aligns with and expands on the International Labour Organization (ILO) and the United Nations (UN)’s ongoing sustainable development agenda to promote decent work and economic growth, eliminate poverty, and reduce inequalities [120, 121]. Most recently, the severe economic and employment crisis triggered by the COVID-19 pandemic brought increased attention to the field of precarious employment research, as reflected in several international reports and calls for equitable solutions to support economic recovery and the protection of workers [122, 123].

### Aims

The objectives of this systematic review are to identify, appraise, and synthesize existing research on the effectiveness of initiatives aiming to or having the potential to eliminate, reduce, or mitigate workers’ exposure to precarious employment conditions and its effects on the physical and mental health, safety, and well-being of workers and their families.

This review is part of a large international project undertaken by the Precarious Work Research Group funded by Forte, The Swedish Research Council for Health, Working Life and Welfare, to understand and address the effects of non-standard employment on worker health.

## Methods

The present review protocol is being reported in accordance with the reporting guidance the Preferred Reporting Items for Systematic Reviews and Meta-Analyses Protocols (PRISMA-P) statement [124, 125] (see PRISMA-P checklist in Additional file 1). This review protocol was registered within the International Prospective Register of Systematic Reviews (PROSPERO) (registration number CRD42020187544). The planned systematic review described in this protocol will be reported following the Preferred Reporting Items for Systematic Reviews and Meta-Analyses (PRISMA) 2020 statement [126]. Any significant protocol amendments will be reported in the publications resulting from this review.

### Eligibility criteria

In order to define the eligibility criteria of the included studies, we use the Population, Intervention, Comparator, and Outcome (PICO) framework, as detailed next.

#### Population

We will include publications focused on workers and their families, adopting the definition of workers used in the Occupational Health and Safety Act, RSO 1990 c.0.1 Ontario, Canada [127], with no geographical limitations. Workers could include (a) females, males, and others; (b) youth (18–24 years of age), adult (25–64 years of age), and senior workers (over 64 years of age); (c) abled and disabled workers; and (d) migrant and non-migrant workers including indigenous populations and ethnic minorities. Family members will be included if they are part of the immediate family, for example dependent and non-dependent children, or if they are part of the extended family, for example siblings, grandparents, and grandchildren.

Publications focused on incarcerated individuals who participate in work projects within a correctional facility or volunteers who work for no monetary payment of any kind are not considered for inclusion.

#### Intervention

We will include initiatives that aim to or have the potential to eliminate, reduce, or mitigate workers’ exposure to precarious employment conditions and/or its effects on the health and well-being of workers and their families, whether or not such initiatives were designed specifically to fight precarious employment or determined to have indirectly affected the realm of precarious employment. For the purpose of this review, an initiative is defined as any type of intervention, programme, strategy, policy, social security, legislation, regulation, legal provision, directive, labour standard, organizational policy, guideline, recommendation, collective agreement or collective contract, plan, experiment, routine, procedure, or practice. To be considered for inclusion, the initiative, the process for its evaluation, and the evaluation outcomes must have been clearly described and the investigation must have included a clear focus on precarious employment conditions or the health of workers in precarious employment and their families. Initiatives will be considered for inclusion no matter their outcome; thus, they could have been deemed successful, unsuccessful, harmful, or inconclusive in their effects on exposure to precarious employment and/or their effects on the health and well-being of precarious workers and their families.

We will exclude initiatives designed to (a) facilitate precarious employment or increase exposure to it; (b) improve workers’ health through individual behavioural change without a focus on precarious employment; (c) improve work performance for workers with disabilities, without a focus on precarious employment; (d) eliminate or reduce workers’ exposure to unemployment; (e) eliminate, reduce, or mitigate the effects of unemployment on health and well-being; and (f) promote workers’ return to work after illness or injury without addressing precarious employment (e.g. initiatives that focus on workplace performance and efficiencies only). We are also excluding studies that assess the impact of worker characteristics on precarious employment since such factors cannot be considered true initiatives. We acknowledge that employment, in any form, is often regarded as the better alternative to unemployment or considered a steppingstone to non-precarious employment. However, for the purpose of this review, we exclude initiatives designed to increase precarious employment among those who are unemployed.

#### Comparator

There are no limitations on comparator. We will include studies that compare the presence and absence of a given initiative or that compare a given initiative to another intervention or set of interventions.

#### Outcomes

The primary outcomes will be changes in (i) the prevalence of precarious employment and workers’ exposure to precarious employment and (ii) the health and well-being of precariously employed workers and their families. There are no secondary or additional outcomes or interest.

Changes in precarious employment rates and workers’ exposure to precarious employment include (a) prevalence of precarious employment; (b) employment insecurity (e.g. contractual relationship insecurity, contractual temporariness, contractual underemployment, and multiple jobs/sectors); (c) income inadequacy (e.g. income level, income volatility); and (d) lack of rights and protection in the employment relation (e.g. lack of unionization, lack of social security benefits and protection, lack of regulatory support and lack of workplace rights including (i) protection against unjustifiable dismissal, (ii) protection from sexual harassment and workplace violence, (iii) protection for whistle-blowers, (iv) unacceptable working practices such as hazardous conditions and other occupational health and safety concerns, and (v) lack of training opportunities).

Changes in health and well-being of precariously employed workers and their families could refer to (a) mental health (e.g. anxiety, depression, post-traumatic stress disorder, burnout, psychoses, substance misuse, and psychological distress), (b) physical health (e.g. physical symptoms and both acute and chronic conditions), (c) safety (e.g. occupational injuries including acute musculoskeletal disorders), (d) well-being (e.g. social support and relationships, sense of belonging, unmet needs, work satisfaction, feelings of community cohesiveness), and (e) health equity (e.g. differences in the previously mentioned groups of health outcomes across class, gender, age, race/ethnicity, migrant status, credentials). Since lack of access to universal health services and universal drug programmes along with the unequal utilization of health services are commonly linked to the creation and maintenance of health inequities, attention will be given to initiatives that promote universal access to such services given their potential to increase health equity.

#### Study design

We will include reports and peer-reviewed primary studies with a qualitative, quantitative, or mixed-methods design. Qualitative studies could have any qualitative methodology including, for example, phenomenology, grounded theory, ethnography, action research, and community research. Quantitative studies could have any experimental or observational study design including, for instance, randomized and non-randomized controlled trials, quasi-experimental, longitudinal, and cross-sectional design. We will exclude editorials, letters to the editor, commentaries, newspaper articles, and discussion papers. Systematic or other types of reviews will also be excluded; however, they will be screened to increase understanding of the depth and breadth of existing research on this topic and to identify relevant studies that could have otherwise been missed through reviews of primary studies alone.

#### Setting

The initiative we are considering could have been implemented at micro-, meso-, or macro-levels, in any region, sub-region, or country, no matter the level of economic development or government unit level.

#### Publication year and status

Studies will be included if published on or after 1 January 2000 and if published in peer-reviewed journals or as grey literature.

#### Language

Only documents with abstracts in English will be considered for inclusion. If relevant sources of information written in languages in which a member of the review team is fluent (Catalan, Danish, Dutch, French, Italian, Norwegian, Romanian, Spanish, and Swedish) are identified based on their English language abstracts, appropriate members of the review team will review them to assess their eligibility for inclusion.

### Information sources

Our sources of information include three academic databases, Scopus, Web of Science Core Collection, and PubMed, along with three institutional databases as sources of grey literature: ILO, European Foundation for the Improvement of Living and Working Conditions, and the Centers for Disease Control and Prevention Community Guide of Evidence-Based Findings, covered from January 2000 onwards. Additionally, the reference lists of eligible studies and research citing the eligible studies will be reviewed to identify further potentially relevant studies. Topic experts and various stakeholders involved in precarious employment research through the Precarious Work Research (PWR) group along with their partners will also be asked for recommendations regarding potentially relevant studies.

### Search strategy and search strings

The search strategy is designed around the intervention component of the PICO framework, along with terms focused on outcomes and evaluation, combined using the Boolean operators AND and OR. The search terms focused on the intervention include variations of the words initiative, intervention, programme, strategy, policy, legislation, regulation, guideline, practice, recommendation, or procedure. Examples of employment-related outcomes include variations of the words precarious, informal, casual, atypical, non-standard, temporary, gig, part-time, and contract, while the evaluation terms are built around words such as evaluation, assessment, appraisal, and measure. Given our interest in a wide range of possible health-related outcomes, health-related ones are not included in the search. Given the wide range of possible meso- and macro-level initiatives that are not necessarily directed at our population, population-related search terms are not included in the search. Although some of the PICO components are not reflected in the search, all studies are screened against the determined eligibility criteria.

The search strategies were designed by the co-authors with advice from three specialized librarians, one at the Karolinska Institute and two at the Karlstads University in Sweden. The librarians are conducting the academic database searches, and the study co-authors are executing the grey literature ones. A draft search strategy for MEDLINE is included in Additional file 2.

### Study records

#### Data management

The study records and data will be managed through a combination of data management, data extraction, and referencing programmes and applications including: Covidence, Zotero, EndNote, Microsoft Teams, Google Forms, and Excel spreadsheets.

#### Data selection and collection

All records identified through various searches will be uploaded through the referencing systems Zotero and EndNote and managed in Covidence. Given the large body of evidence screened, the title and abstract screening of each study will be done independently by one reviewer, after implementing several strategies to ensure decision-making consistency across reviewers. These strategies include clear guidance regarding inclusion/exclusion criteria, abstract screening training, pilot testing the screening process several times to ensure that all reviewers have the same understanding of the inclusion/exclusion criteria used to guide the selection of studies, and bi-weekly meetings to address questions. As part of the pilot testing, initially, reviewers will screen the same subsets of records individually and then compare decisions. Once the inclusion/exclusion decisions among the reviewers are consistent, reviewers will proceed to screen the remaining titles and abstracts independently. In addition, any record that a reviewer will be unsure about will be marked as a “maybe” and will be screened subsequently by at least two other reviewers to determine whether it should be selected for full-text review.

A data extraction template was created to facilitate the capturing of specific data, including study name and author(s), study design, dimension of precarious employment addressed, purpose and description of initiative, target population(s), outcome(s), evaluation results, country, industry/occupation, the source(s) of funding, and other potential conflicts of interest. The data extraction template is currently being piloted by the reviewers involved in the title and abstract screening and will be adjusted as needed. All studies recommended for full-text review will be divided up among pairs of reviewers, who will complete the data extraction for the included studies independently. For studies excluded at the full-text review stage, only a few sections of the form will be completed, capturing enough information to document reason(s) for exclusion. A third reviewer will read through the extracted data for each study to determine whether to confirm the decision. Any disagreements will be discussed with the other reviewers, until consensus is reached. A flow diagram of the papers identified for inclusion will be created using the PRISMA 2020 statement [126].

### Risk of bias in individual studies

The included studies will be critically appraised for risk of bias and quality by two reviewers independently. All disagreements will be discussed and resolved in consultation with a third reviewer. The results of the risk of bias appraisal will be shared using a combination of tables, figures and narrative comments to outline the strengths and limitations of the included studies, both individually and as a whole.

Our team will use the Critical Appraisal Skills Programme (CASP) Checklists to assess qualitative and quantitative studies [128]. A study will be labelled as low-quality if the answers to two or more questions in sections A and B of the tool are “Can’t Tell” or “No”. The Mixed Methods Appraisal Tool (MMAT) will be utilized to assess mixed-methods studies [129]. As suggested by the MMAT tool’s creators, each study will be rated for each relevant category and a detailed presentation of the rating of each criterion will be shared. No studies will be excluded based on the critical appraisal results; however, this information will be clearly stated to support the interpretation of the included studies’ findings and confirm the validity of our systematic review’s outcomes and conclusions.

### Data analysis and synthesis

We will present the results of this systematic review using a combination of structured narratives and summary visuals. The within and between heterogeneity of all primary studies—qualitative, quantitative, and mixed-methods—and their findings will be synthesized narratively, using text, tables, and figures. Although we include primary studies with quantitative and mixed-methods research designs, given the wide range and diversity of interventions, outcomes, and evaluation methods, meta-analysis would not be appropriate and will not be performed. However, to ensure transparency in our narrative synthesis reporting we will follow the Synthesis Without Meta-analysis (SWiM) reporting guideline to describe in a structured manner the (i) methods and findings used in the primary studies, (ii) grouping of studies, and (iii) synthesis approaches and their limitations [130].

For each included study, we will report specific information, including study name and author(s), study design, dimension of precarious employment addressed, purpose and description of initiative, the level of the initiative (macro/meso/micro), target population(s), primary outcome(s), evaluation results, and country. We will clearly specify the ways in which the outcomes are measured (e.g. relative risks, odds ratios, risk difference, or in qualitative terms, depending on each initiative assessed). When available, information specifically related to women, youth, racialized, ethnic, or other minority subgroups, foreign-born, lower-educated, disabled, and informal workers will be also included, as these subgroups are at disproportionate risk of experiencing precarious employment. Similarly, information related to employees who transitioned from standard to non-standard employment will also be captured given specific policy interests in the increase of precarious employment over the last several decades among this subgroup. Moreover, whenever mentioned in the primary study or report, factors that may have contributed to an initiative’s success or failure, and factors that could increase the transferability of an initiative to other settings, will be captured. To speed up the process of knowledge translation, increase transferability of findings, and facilitate their dissemination into practice settings, results may also be grouped into separate publications according to the categories mentioned in this section.

### Confidence in cumulative evidence

In order to assess the strength of the evidence identified through our systematic review, we will adopt the Grading of Recommendation, Assessment, Development and Evaluation (GRADE) approach [131, 132]. This will allow us to rate the quality of the evidence according to five considerations: study limitations, effect consistency, imprecision, indirectness and publication bias and, as needed, downgrade or upgrade the evidence presented in the included studies [131]. The grading process will be performed independently by two reviewers and the results will be shared using tables that summarize confidence across and between studies.

## Discussion

Our systematic review will identify, assess, and synthesize available information on evaluated initiatives to eliminate, reduce, or mitigate workers’ exposure to precarious employment and its effects on the health and well-being of workers and their families. This review seeks to fill a significant gap in existing knowledge on strategies to counteract precarious employment and its harmful effects on health and well-being that continues to exist despite a recent increase in precarious employment research. This review’s findings will provide stakeholders interested in addressing precarious employment an evidence base on solutions that have been undertaken and the potential for adaptation of these to their unique contexts. The findings will also facilitate an increased understanding of research gaps and potential ways to address them. In addition, this systematic review’s findings will complement the research outputs of the broader Precarious Work Research (PWR) Group.

Given the complexity of this research topic and the broad scope of this systematic review, a range of topic experts provided feedback on the creation of the review’s protocol and are currently involved in conducting the review. The co-authors’ extensive expertise relevant to this topic will be instrumental in addressing any conceptual and operational issues arising during the completion of the review. Thus far, we acknowledge several potential limitations. First, given the large body of evidence screened and the relatively limited resources, the title and abstract screening of each study will be done independently by one reviewer only. To address this potential constraint, we will implement several strategies to ensure decision-making consistency across reviewers. Second, given the need to balance scope with available study personnel, we limited our literature search to only three academic databases and three sources of grey literature and; for this reason, it is possible that we will miss relevant studies that fit our inclusion criteria that are indexed elsewhere. To address this limitation, in collaboration with our librarians, we decided to use two interdisciplinary academic databases with the least overlap in included sources. In addition, we will hand-search both the reference lists of eligible studies and research citing the eligible studies, in addition to consulting several topic experts and stakeholders for their suggestions on relevant studies. Once this review is complete, we plan to expand on this work with a realist systematic review of relevant initiatives. This will allow us not only to identify additional initiatives but also to learn more about which initiatives work for whom and under what circumstances, in order to enhance the applicability of our findings and enable their implementation in different contexts. Our practical decision to exclude studies without an English abstract constitutes another limitation, which we tried to overcome by including a range of non-English publications as long as they have an English abstract and they are written in a language in which a member of the review team is fluent (Catalan, Danish, Dutch, French, Italian, Norwegian, Romanian, Spanish, and Swedish).

The COVID-19 pandemic and resulting global economic crisis have highlighted the risks that decades of increases in precarious employment have created for workers and society as a whole. Our systematic review, with its focus on strategies to address these risks, is therefore extremely timely and relevant. Our work not only aligns with the sustainable development agenda endorsed by the ILO and UN to promote decent work and economic growth, eliminate poverty, and reduce inequalities (ILO, 2018; United Nations, 2019), but it also responds to several recent calls by international organizations and funding agencies for research focused on finding equitable solutions to support economic recovery and the protection of workers [122, 123].

It is our hope that the findings of this systematic review will support the work of international labour organizations, governments, labour unions, employers, workers, and other stakeholders. It should provide them with evidence on effectiveness of strategies to consider for their unique contexts in their fight against precarious employment and the promotion of workplace environments that enable workers to thrive. This may foment the use of findings which exist, and prompt the testing of more initiatives once knowledge gaps are clarified. We will summarize the research knowledge gaps on this topic, as identified through our review, and make recommendations to address them.

## Supplementary Information

**Additional file 1.** PRISMA-P 2015 Checklist.

**Additional file 2.** MEDLINE (Ovid) Search Strategy.

## Data Availability

Not applicable

## Abbreviations

CASP: Critical Appraisal Skills Programme Checklist
ILO: International Labour Organization
PICO: Population, Intervention, Comparator, and Outcome Framework
PRISMA-P: Preferred Reporting Items for Systematic Review and Meta-Analysis-Protocol
PROSPERO: The International Prospective Register of Systematic Review
PWR: Precarious Work Research
UN: United Nations
