# Supplementary material for: Initiatives addressing precarious employment and its effects on workers’ health and well-being: a protocol for a systematic review
Source: Syst Rev. 2021 Jun 30;10:195. doi: 10.1186/s13643-021-01728-z (PMC8244669; doi:10.1186/s13643-021-01728-z)
Supplement: Supplementary file 2 — Additional file 2. MEDLINE (Ovid) Search Strategy. [file 13643_2021_1728_MOESM2_ESM.docx]

MEDLINE (Ovid)

| Interface: Ovid MEDLINE(R) and Epub Ahead of Print, In-Process, In-Data-Review & Other Non-Indexed Citations and Daily  Date of Search: 17 May 2021  Number of hits: 1,769  Comment: In Ovid, two or more words are automatically searched as phrases; i.e. no quotation marks are needed  The interface searches the same material as via PubMed. | Field labels   - exp/ = exploded MeSH term - / = non exploded MeSH term - .ti,ab,kf. = title, abstract and author keywords - adjx = within x words, regardless of order - * = truncation of word for alternate endings |
| --- | --- |
| Database(s): **Ovid MEDLINE(R) and Epub Ahead of Print, In-Process, In-Data-Review & Other Non-Indexed Citations and Daily**1946 to May 14, 2021 Search Strategy:   \| **#** \| **Searches** \| **Results** \| \| --- \| --- \| --- \| \| 1 \| ((precari* or informal* or casual or atypical or non-standard or tempora* or gig or part-time or project-based or sub-contract) adj3 (work* or employ* or labour)).ti,ab,kf. \| 6775 \| \| 2 \| exp Employment/ \| 90126 \| \| 3 \| (precari* or informal* or casual or atypical or non-standard or tempora* or gig or part-time or project-based or sub-contract).ti,ab,kf. \| 616678 \| \| 4 \| 2 and 3 \| 3784 \| \| 5 \| 1 or 4 \| 8775 \| \| 6 \| (initiative* or intervention* or program* or strateg* or polic* or social security or legislation* or regulation* or legal provision* or directive* or labour standard* or organizational polic* or guideline* or recommendation* or collective agreement* or collective contract* or union agreement* or plan* or pilot or test* or trial* or experiment* or routine* or practice* or procedure* or childcare).ti,ab,kf. \| 11348508 \| \| 7 \| exp Labor unions/ \| 9020 \| \| 8 \| exp Child Care/ \| 20373 \| \| 9 \| exp Social Security/ \| 8306 \| \| 10 \| exp Jurisprudence/ \| 208023 \| \| 11 \| Organizational Policy/ \| 14346 \| \| 12 \| or/6-11 \| 11506600 \| \| 13 \| (evaluat* or assess* or apprais* or measure*).ti,ab,kf. \| 8134236 \| \| 14 \| 5 and 12 and 13 \| 2118 \| \| 15 \| exp Program evaluation/ \| 78444 \| \| 16 \| 5 and 15 \| 105 \| \| 17 \| 14 or 16 \| 2158 \| \| 18 \| limit 17 to yr="2000 -Current" \| 1834 \| \| 19 \| editorial.pt. \| 567322 \| \| 20 \| 18 not 19 \| 1833 \| \| 21 \| (english or italian or spanish or french).lg. \| 29190229 \| \| 22 \| 20 and 21 \| 1769 \| | |
